# Supplementary material for: The interactome of intact mitochondria by cross-linking mass spectrometry provides evidence for coexisting respiratory supercomplexes
Source: Mol Cell Proteomics. 2017 Dec 8;17(2):216–32. doi: 10.1074/mcp.RA117.000470 (PMC5795388; doi:10.1074/mcp.RA117.000470)
Supplement: Supplemental Data [file supp_RA117.000470_133922_0_supp_23978_fzffwf.zip › spectra_annotation/mito_spectra_annotation/219-1-17-1-3-1.pdf]

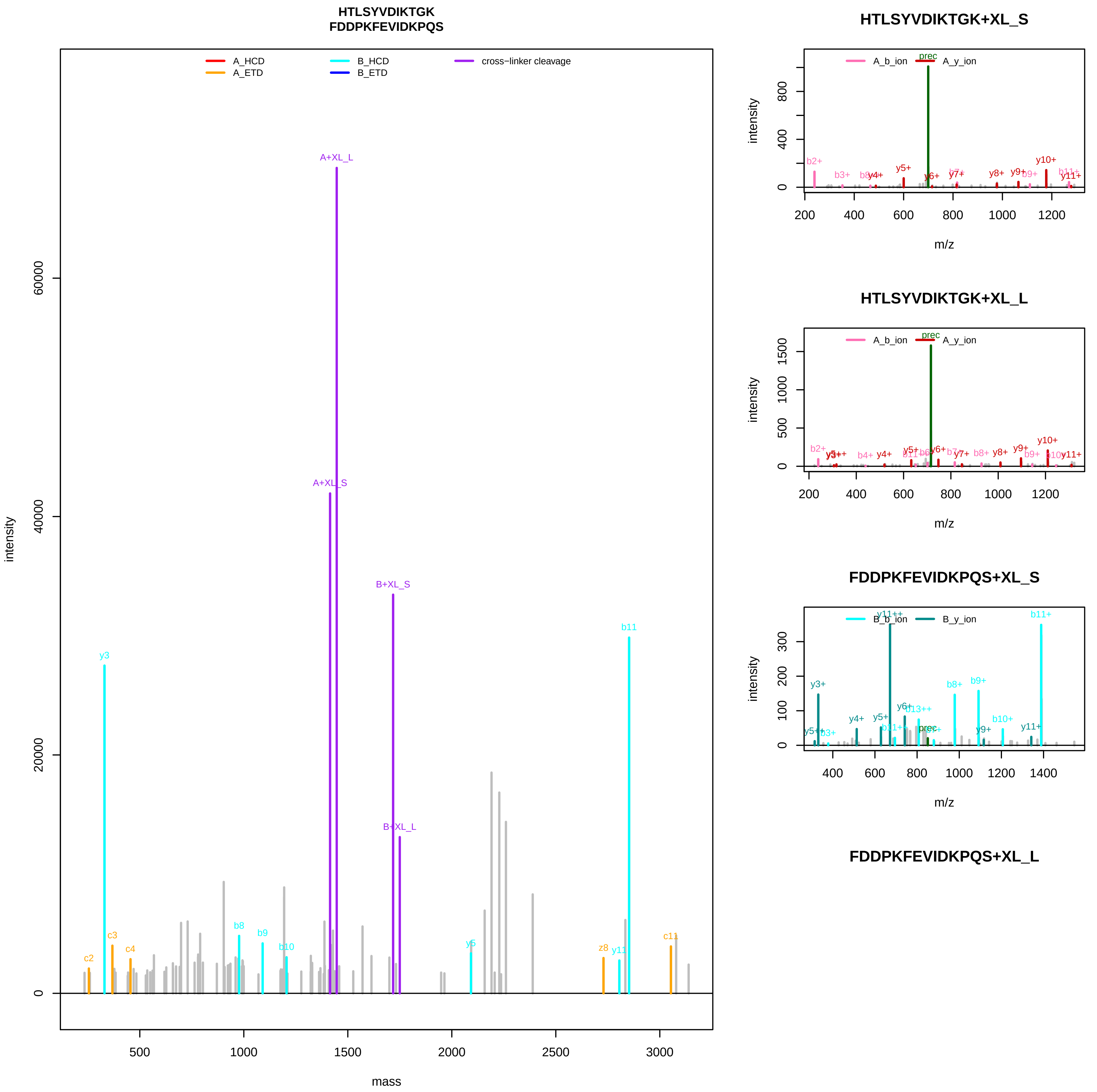

HTLSYVDIKTGK
FDDPKFEVIDKPQS
A_HCD
A_ETD
B_HCD
B_ETD
cross−linker cleavage
A+XL_L
A+XL_S
B+XL_S
B+XL_L
y3
b11
c2
c3
c4
b8
b9
b10
y5
z8
y11
c11
intensity
mass
0
20000
40000
60000
500
1000
1500
2000
2500
3000
HTLSYVDIKTGK+XL_S
A_b_ion
A_y_ion
prec
b2+
b3+
y4+
y5+
y6+
y7+
y8+
y9+
b9+
y10+
y11+
m/z
HTLSYVDIKTGK+XL_L
b4+
b7+
b8+
FDDPKFEVIDKPQS+XL_S
B_b_ion
B_y_ion
y11++
b11+
y3+
b8+
b9+
y4+
y5+
y6+
b13++
b10+
y9+
y11+
FDDPKFEVIDKPQS+XL_L
